# Human grasping database for activities of daily living with depth, color and kinematic data streams

**DOI:** 10.1038/sdata.2018.101

**Published:** 2018-05-29

**Authors:** Artur Saudabayev, Zhanibek Rysbek, Raykhan Khassenova, Huseyin Atakan Varol

**Affiliations:** 1 Nazarbayev University, School of Science and Technology, Astana Z05H0P9, Kazakhstan

**Keywords:** Biomedical engineering, Electrical and electronic engineering

## Abstract

This paper presents a grasping database collected from multiple human subjects for activities of daily living in unstructured environments. The main strength of this database is the use of three different sensing modalities: color images from a head-mounted action camera, distance data from a depth sensor on the dominant arm and upper body kinematic data acquired from an inertial motion capture suit. 3826 grasps were identified in the data collected during 9-hours of experiments. The grasps were grouped according to a hierarchical taxonomy into 35 different grasp types. The database contains information related to each grasp and associated sensor data acquired from the three sensor modalities. We also provide our data annotation software written in Matlab as an open-source tool. The size of the database is 172 GB. We believe this database can be used as a stepping stone to develop big data and machine learning techniques for grasping and manipulation with potential applications in rehabilitation robotics and intelligent automation.

## Background and Summary

Understanding and replicating human hand grasping and manipulation have been long-running objectives of researchers from multiple disciplines. Qualitative improvements in both will trigger rapid advances in a large number of domains including industrial automation, humanoid robotics, medical rehabilitation and prosthetics. Contemporary anthropomorphic multi-fingered robot hands have achieved levels of dexterity, agility and object manipulation comparable to that of human hands^1,2^. Successful in narrow task-specific applications, these artificial systems, however, still fail to deliver the full functional range of a human hand. While novel sensors^3^, actuators and batteries have been continuously driving the progress, control of artificial hands remains non-trivial and challenging. Control systems link the sensing and actuation components of a robot hand. In prosthetics, control inputs originate in a user, and one common way of acquiring the intention is based on electromyography^4^, where electric signals from the arm musculature are classified into one of set of discrete grasp patterns. Implementation of a grasp is then executed by hardware-specific controllers which generate trajectories for different robot hand segments. Rather limited in their versatility and generalization capabilities, current control systems only enable a limited set of operations, such as opening, closing and several programmable actions enabled by straightforward sequential controllers^5^.

Grasp-synthesis models depend on understanding the mechanistic complexity of the human hand, its sensory-motor control loop, and the relation of grasp taxonomy to object geometry. Emergence of data-driven motion planners emphasized the need for multi-modal high-fidelity data enabling thorough analysis of human grasping. Among data acquisition techniques, glove-based systems deliver information straight from a human hand with high spatial resolution^6^. The widespread use of RGB-Depth cameras facilitated fine-grained activity analysis including hand tracking and hand-object interaction^7^. Conjunction of multiple sensing modalities, e.g. augmentation of RGB-Depth data into optical hand motion tracking system^8^ proved superior to single mode sensory data. Observation of human hand activity is not limited only to limb tracking. The human body consists of connected segments, and the kinematic configuration of the human body was used to predict a dominant hand location^9^. Ye *et al.*^10^ also demonstrated that hand motion can be predicted from upper body inertial measurement data.

Generation and annotation of a versatile database for grasp planning is an arduous task. Researchers usually seek balance in data representation and focus on one of the components of the grasp planning, e.g. target object shape information. The Columbia Grasp Dataset^11^ exemplifies this tendency, where Goldfeder *et al*. introduced a large labeled dataset of scaled 3D object models and baseline grasps feasible for each of them. They then classified unknown objects to the most similar ones in terms of geometric shape. Another common approach is video recording of human activity and subsequent annotation into discrete actions. Bullock *et al*.^12^ used a head-mounted video camera to record regular work activities of two housekeepers and two machinists. The authors presented the 27.7 hours-long Yale Human Grasping dataset with annotated object, grasp and task types. The dataset was instrumental for revealing the frequencies of common grasps in household and machine shop settings^13^. Recent projects on human hand utilization use novel sensors and expand the knowledge application domain. Kleinhans et al. reported on an ongoing project which acquires RGB-Depth images along with geometric description of objects to create a database of successful and failed grasps^14^. To benefit from crowdsourcing in data generation, Kent and Chernova introduced a user-friendly and intuitive web-based interface for grasp learning by demonstration^15^.

In this work, we introduce a multisensor hand grasping dataset which expands the scope of the observations and human activity range compared to the prior works. The main contribution of our paper is in the dataset generated using three sensing modalities, namely RGB camera, depth sensor and upper body inertial motion capture suit. We conducted experimental sessions for data gathering with multiple human subjects executing activities of daily living (ADLs). These activities included food preparation, housekeeping, folding clothes and ironing. Subsequently, the data streams were synchronized and grasps were annotated. Based on our survey of related works, we state that the presented dataset is structurally correct and useful for multi-faceted analysis and application in grasp planning, prediction and evaluation. Moreover, it can be employed to test object recognition and classification algorithms in machine learning and computer vision.

## Methods

### Study Participants

**Figure 1:**
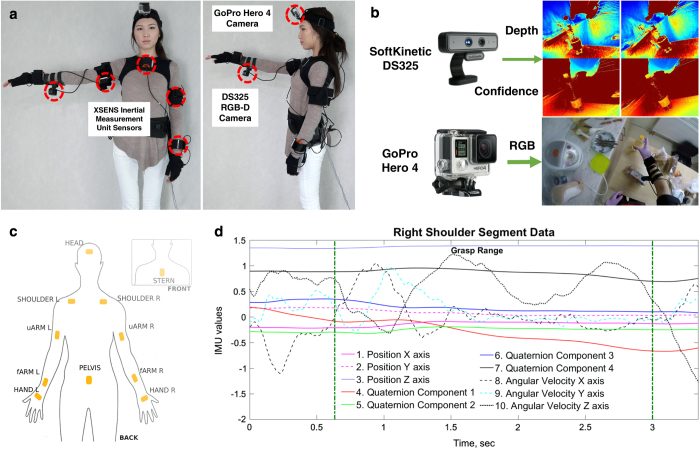
Experimental Setup and Data Acquisition. (**a**) User wearing the hardware setup with major components (Participant consented to the use of her photo in this paper). (**b**) Example snapshots from RGB and Depth sensors. (**c**) Schematic depiction of IMU distribution in upper body XSENS suit configuration. (**d**) Example capture of signals acquired from Right Shoulder segment.

The announcement inviting participants for our study was distributed throughout the Nazarbayev University campus. 13 participants (five females and eight males) expressed their intention to participate in the experiments. Their ages ranged from 19 to 42. At the moment of the experiments, all participants reported no known hand or arm injury, or other health issues which could affect their performance. All 13 participants were right-handed. Before engaging in experiments, each subject was comprehensively briefed about the procedure, introduced to the experimental hardware and informed of any potential risks. Additionally, we provided a written description of the experiments and required participants to sign an informed consent form. The study and experiments were carried out in accordance with principles of the Declaration of Helsinki^16^, and approved by the Institutional Research Ethics Committee of Nazarbayev University, Kazakhstan. The study participant wearing the data acquisition setup in Fig. 1a consented to have her picture included in this publication.

### Procedure

“Activities of Daily Living” is a commonly used term in rehabilitation and occupational therapy, referring to set of everyday tasks critical for unassisted living. Recently, the term gained wider usage amongst the robotics community with similar connotations–to enable evaluation of an artificial system’s performance for daily tasks. ADLs were then sub-categorized to accommodate different application domains. Domestic activities of daily living (DADL) encompass tasks regularly performed in human living environments, e.g. housekeeping and food preparation. Extradomestic activities of daily living (EADL) cover tasks systematically performed outside of home^17^, such as driving or shopping.

Our experimental procedure was comprised of three sessions. Each was designed to encompass a wide range of common activities performed at a household, e.g. cleaning routines, dealing with cutlery, food items, clothes and special equipment such as an iron. Each experiment hence included DADL tasks, which were generalized to food preparation, housekeeping and ironing/clothes folding activities. The exact sequence of DADL experiments is as follows:

- cooking breakfast and cleaning the involved kitchen areas
- housework activities, e.g. wiping the dust and vacuum cleaning
- clothes folding and ironing

Experiments took place in a two-room apartment. All activities were performed in a large room which combines the living room and the kitchen. Before the experiment, subjects were introduced to the general outline of the three experiments, provided with high-level task characterization and thorough instructions about the hardware involved. The duration of each of the experiments and approach to its accomplishment were decided by participants (e.g. subjects were asked to cook breakfast, but decided themselves on what the meal will be and which cutlery to use). Each participant then provided age, gender and a dominant hand information, signed a consent form and were given time to feel themselves comfortable with the setup. Participants were advised to stop the experiment whenever they feel uncomfortable, or with the first sign of fatigue. Two subjects decided to conclude the procedure before starting the laundry activities, with the other eleven finishing the full DADL routine.

#### Data acquisition and data quality

It took each subject between 30–50 min to finish data acquisition. Total duration of experimental data obtained is around 9 h.

During data acquisition, we encountered instances of dropped depth frames and connection loss with the Xsens motion capture suit. Both occurred sporadically, however, missing data in the two data streams caused mismatch and time shift in data frames, which convoluted the synchronization of sensor channels. The problem aggravated with the increased duration of an experiment recorded in a single-shot, i.e. the number of frame shifts and data mismatches increased and were harder to systemically identify. To tackle this issue, we decided to record experiments in time-limited chunks (up to 10 min sessions). This allowed us to reduce the number of dropped depth frames and broken connections with the motion capture setup (by approximately 90%). Hence, the discrepancy between matching events in RGB and depth data was reduced. Only in two 10-minute chunks, the connection with the motion capture suit was lost causing missing IMU data (subject 7 – Data Segment #5 and subject 9 – Data Segment #1), these instances were denoted in the dataset summary spreadsheet (Data Citation 1) submitted along with the manuscript. Importantly, annotating discrete and compact data chunks proved more convenient to manage.

Before the beginning of an experimental session, the subjects were notified that data acquisition would be conducted in approximately 10 min chunks and they would be notified by the experimenter prior to this time instant, such that when they are notified by the experimenter, they are instructed to conclude their current activity but not to start a new one. During the experiments, the 10-minute constraint was regulated by the experimenter. Towards the 10-minute mark, the subjects would be notified and asked to halt their current activity so to prevent mid-action cuts. With the new recording, subjects commence their current action and proceed. Additionally, operators could terminate the recording intentionally, prior to the expiration of 10 min. This happened when subjects were caught in extended idle states – for example waiting for food in the pan to reach a certain condition.

### Data Acquisition Setup

During the experiments, we performed simultaneous acquisition of: (i) Video material from GoPro Hero 4 camera, (ii) Depth and confidence images from SoftKinetic DS325 RGB-Depth camera, and (iii) Upper-body inertial motion data from Xsens MVN motion capture suit (see Fig. 1).

#### Video

A GoPro Hero 4 Silver action camera was attached on top of the head of a participant facing forward slightly inclined downward to capture subject’s hand operational area. The weight of the camera is 83 grams. It is 59 mm in length and 41 mm in height. The data was acquired at 30 frames per second with 1280×720 pixels resolution, and recorded to the camera’s internal storage.

#### Range data

Depth images were acquired using RGB-Depth camera DS325 (SoftKinetic). We located the depth camera to the middle of the ventral-side of the right forearm of a subject. Depending on the forearm size of a subject, the distance between the radiocarpal joint and the camera was in the range of 11–15 cm making the distance between the depth camera and the center of the palm 16 to 22 cm. The camera attached to a 3D-printed forearm brace has a 6 cm offset from the arm surface to the camera sensor. The setup is aligned with the arm frontal plane, however the anatomical variance in forearm thickness results in a slight inclination towards the hand workspace region (around 5 degrees with the arm frontal plane). This configuration enabled us to capture most of the grasping activity with the camera field of view of 74°×58°×87° (H×V×D) and the nominal working range of 0.15 – 1 m.

Additionally, the DS325 camera provides a confidence image along with each depth frame. Confidence images express the magnitude of infrared light inflicting on the detector, hence expressing the level of “reliability” of depth measurement in the corresponding pixel of a depth map. Both depth and corresponding confidence images were recorded with 320×240 pixels resolution at 30 frames per second frame. The camera was connected to the HP EliteBook 8560W laptop (Windows 7 operating system, Intel Core i7 processor), which was used for both acquisition and storage of range data.

#### Inertial motion data

Upper-body inertial motion data was recorded using an Xsens MVN suit^18^. The suit is comprised of 17 sensors dividing the body into 23 segments and 22 joints. In this work we only used the upper body configuration of the Xsens MVN, which includes 11 sensors and omits segments and joints related to legs, feet and toes. Each sensor has a 3-axis gyroscope, accelerometer and magnetometer. The sensors are placed on a user’s body as indicated in Fig. 1c. The processed raw data from these sensors enables derivation of position, angular velocity and quaternion data for each body segment. With position and angular velocity expressed as triplets with respect to *x-*, *y-* and *z*-axis, and four quaternion components for segment orientation, there are ten records per each of the upper body segments. Data from the inertial measurement unit sensors was acquired at 120 frames per second and transmitted to the HP EliteBook 8560W laptop (Windows 7 operating system, Intel Core i7 processor) via a USB connection.

### Data Annotation

#### Grasp definition

After acquisition, the experimental data was visually analyzed for annotation and mapping by human experts. To avoid conceptual ambiguity, we adopted the definition of the grasp as established by Feix *et al*.^19^.

*“A grasp is every static hand posture with which an object can be held securely with one hand.”*

This definition excludes any intrinsic in-hand motion and object manipulation. We further identified discrete grasp actions, similar to the concept of elementary grasp actions employed by Vergara *et al*.^20^ the starting point of each grasp action was established when a human annotator detects contact of the subject’s hand with an object. The end point of the action was established when the subject released an object or performed another grasp.

The rare cases when a grasp is followed by another grasp without the release of the initially grasped object are handled using the notion of grasp ‘sequence number’. These sequence numbers are included in the annotation data provided as an Excel spreadsheet (seqNum column) along with the dataset. We record every grasp and assign to it a sequence number. The end of the grasp is identified at the moment when an object is released or in-hand repositioning occurs and a new static hand posture is formed.

As an example, consider the case when a grasp is engaged (normally using fingers) while the object acquired during the previous grasp is still in hand (e.g. fixed in palm). At such instances, we ended the preceding grasp (let’s refer to it as grasp_1_) and denoted the start frame of a new grasp with the new sequence number (grasp_2_). The initially grasped object in all observed cases was repositioned in hand or manipulated. If the subject finishes the grasp_2_ and re-grasps the object from the grasp_1_ after manipulation, it would result in a new record grasp_3_.

#### Annotation and synchronization

Grasp annotation was performed by observing RGB video from the action camera stream. The grasp start frame was identified as the moment of contact of subject hand with an object. In case of occlusion (present in less than five percent of the grasps), the start frame is selected by estimation based on the observation of the depth frames. Grasp end frame is recorded when the subject releases an object, or transits to a different static grasp (which also becomes the start frame of the subsequent grasp). Video, range and inertial motion data were recorded asynchronously, and segments of each data stream started at different time instants. Manual synchronization of RGB, inertial motion and depth data was performed using a custom written software. Specifically, grasp events identified in the action camera videos were mapped to the corresponding frames in the depth and inertial motion data. The start and end frames of each grasp were then recorded, respectively. Additionally, we provide a graphical user interface which allows annotation and visualization of all three streams and the means to access specific frames in each of the channels. The depth stream was visualized using the Jet color map of Matlab (see Fig. 1b). Only the right-hand grasps (dominant hand of each participant) were considered.

Grasp annotation and synchronization were performed by two researchers with engineering backgrounds. The data was split into two roughly equal parts and annotated separately. Subsequently, the annotators reviewed the results together with a third expert to resolve ambiguous grasps with majority voting. Timeline outside of the grasps was left untagged. Total number of grasp actions annotated in this study is 3826.

#### Grasp taxonomy and assumptions

To organize and encompass the complexity of the high number of grasping patterns, it is common to arrange them into a taxonomy^21^. This facilitates structural and functional relation analysis of grasps, and provides researchers with powerful analytical tools. As an example of the latter, Feix *et al.*^19^ performed hierarchical structuring of 33 grasps acquired from the literature and reduced them to 17. Quantitative narrowing of the grasp space while retaining its functional gamut might reduce the complexity of artificial limbs with minimal functionality loss.

Grasp taxonomy reduction offers a tradeoff between simplification and representation capability specific to the application domain and/or use case. In our work, we wanted to provide a wider selection of grasps, and the corresponding sensory and statistical data. This way, we intended to make the dataset useful for researchers from multiple domains. Especially, we considered the field of prosthetics, and development of controllers for artificial hands. It is essential for this domain to have major non-prehensile actions to be included into the taxonomy. Non-prehensile hand movements do not result in force or form closure. They might utilize the fingers or the hand in general, however, they do not result in an object acquisition or capturing by the hand^22^. Therefore, any hand movement which aimed at pushing (button press, moving objects on a surface by pushing them with an open palm, pushing a door to close it, etc.) or lifting (holding an item on a palm without achieving a form closure) were considered as push and lift grasp types, correspondingly.

**Figure 2:**
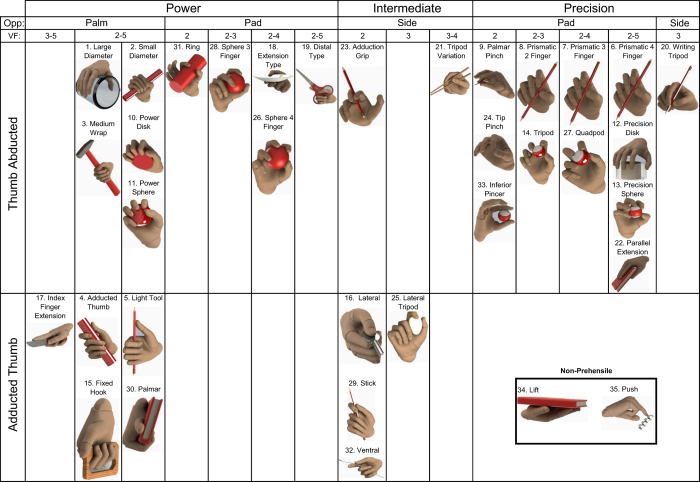
Grasp Taxonomy. Taxonomy of grasps classified and depicted according to thumb position, hand and finger configuration.

We followed the original non-reduced taxonomy provided by Feix *et al*.^19^ In addition to these 33 grasps, we added two common non-prehensile configurations – *push* and *lift*, obtaining 35 grasps in total (see Fig. 2). Furthermore, we employed two assumptions from their work^19^ for the grasp annotation. Specifically, we omitted *bimanual* tasks, which are possible only with the application of two hands (e.g. two-hand bedsheet folding), and we applied the notion of grasp sequence number to deal with sequence of grasps transitioning through in-hand manipulation.

### Code availability

The Matlab code used for annotation of the experiments and assigning grasp labels is available at https://github.com/zhanibekrysbek/Annotation-software-of-Human-Grasping-Database. Furthermore, we are providing the Matlab code allowing visualization of data from the three data streams – RGB, depth and inertial motion capture suit. Visualization software is available at https://github.com/zhanibekrysbek/visualization_software_human_grasping_database. Both of the repositories are accompanied with detailed usage instructions.

## Data Records

Data records introduced in this paper along with the dataset summary and the grasp annotation file are available through the Figshare repository (Data Citation 1). Dataset summary spreadsheet provides information on human subjects’ gender and age, as well as it locates relevant files for each participant in the dataset. The overall size of the data is 172 GB. It was arranged into five main directories and archived: three archived files for depth data (Depth_1.zip, Depth_2.zip and Depth_3.zip), RGB (GoPro.zip) and inertial motion data (Xsens.zip). Each of the five directories consists of human subject data of the corresponding nature (i.e. depth, RGB and inertial motion) acquired during experiments. We de-identified human subjects, and assigned unique identifiers for the directory names. For instance, a folder named ‘1. Subject 1’ in the Depth_1 directory will contain all depth stream data acquired from subject 1 during experiments. ‘Subject 4’ folder in the GoPro directory will contain all video material collected with GoPro action camera from subject 4 during experiments. As it was mentioned in the Procedure section, there were examples of intentional termination of experimental activities. For example, subject 7 stopped the experiment by their own will before commencing laundry activities, hence the corresponding subject files only contain data for food preparation and housekeeping exercises.

We selected an example single episode from the entire dataset – an excerpt of approximately 10 min duration with RGB, depth and motion data records. The episode is archived and submitted along with the dataset as ZIP archive (***sample_record.zip***). The size of the sample record is around 3.2 GB.

### Missing data

There are instances of missing inertial motion capture data. Due to communication errors, some packages were dropped during transmission from the Xsens motion capture suit, making an entire record segment inconsistent. These segments were not included in the dataset, and the corresponding fields in the Data Summary spreadsheet (Data Citation 1) are denoted as ‘Missing Data’. Additionally, depth stream also experiences missing data. For example, the system would acquire 28 frames instead of 30 frames per second. The direct implication of such behavior is the shift in frame numbers for grasp intervals with RGB and inertial motion streams. The magnitude of such shift was minimized by limiting the experiments interval to less than 10 min, and then neutralized by synchronization and manual mapping. In the annotation file, the difference between start and end frames for grasps in RGB and depth modes can be observed. In general, this missing data does not incur any significant information loss and is not a limiting factor.

### Raw data

Five main data directories contain action camera videos (RGB), depth and confidence videos and inertial motion data. Action camera videos are stored as Audio Video Interleaved (AVI) files, depth and confidence images in the Motion JPEG 200 (MJ2) format, and inertial motion data as comma-separated values (CSV) files. As mentioned in the Methods section, the length of the experiments varied across participants and acquisition was performed in segments of no longer than ten-minute duration. This way, for every subject, there are different number of files available.

Data acquired from the upper-body configuration of the Xsens motion capture suit was arranged into 227 data columns and stored as a CSV file. The first 150 columns contain the data from 15 segments including Pelvis, Right Hand, Right Forearm, Right Upper Arm, Right Shoulder, Neck, Head, etc. Each consecutive ten fields out of 150 represent three position, four orientation quaternion and three angular velocity values of each segment. Subsequently, there are 77 columns, which contain three gyroscope and four quaternion values for each of the eleven sensors of the upper body suit such as Pelvis, Head, Right Hand, Right Upper Arm, etc.

### Annotation records

The annotation information is provided as a CSV file in the root of the Figshare dataset (Data Citation 1). There are 12 columns in the annotation file. The file indicates the ID of the grasp and the participant to whom it belongs. In addition, the file identifies the grasp type as identified from the taxonomy, task type as annotated by human experts and start and end frames of the episode to locate them in the GoPro video files. Finally, the annotation describes the grasp properties including opposition type, power level and thumb position. From first to last, the columns represent the values for *Grasp ID, Participant ID, Grasp Type, ADL, Video File Name, Start Frame GoPro, End Frame Gopro, Opposition Type, Power level, Virtual Fingers, Thumb configuration, Duration(in sec), Start Frame Depth, End Frame Depth, Sequence Number*.

## Technical Validation

In this section, we provide statistical evaluation of the data acquired from 13 participants during almost nine hours of experiments annotated by human experts. We first analyze the frequency and duration data of the grasp dataset. Subsequently, we compare subjects’ experimental data during specific ADLs, and outline average grasp statistics for each of the DADLs. In order to validate the acquired data, we compare our results with the prior literature (Vergara *et al.*^20^ and Bullock *et al.*^23^), where authors evaluated statistical usage of grasps in different activities. While their grasp taxonomy and engaged ADLs differ from ours to some extent, there are many commonalities which can be drawn to bolster the validity of our data.

Out of the 3826 grasps in our dataset, 2922, 564 and 340 grasps were performed during food preparation, housekeeping and laundry activities, respectively. Table 1 illustrates the breakdown of frequency and duration of all grasps given in the context of the ADLs. Out of 35 grasps defined in the taxonomy, three grasps were not employed by the participants and not recorded during the experimental session. These are the distal type, tripod variation and ring. Therefore, Table 1 contains 32 grasps with nonzero frequencies. Sorted in the descending order by total duration, Table 1 reveals that the duration of the top ten grasps accounts for 74% of the total duration, which agrees with Bullock *et al*.^23^, where the duration of the top ten grasps is around 80% of total duration for a similar taxonomy. The top two grasps in our dataset are index finger extension (IndFE) and adducted thumb, with around 17 and 12% of the total grasp duration, respectively.

### Distribution of power, intermediate and precision grasp types

**Figure 3:**
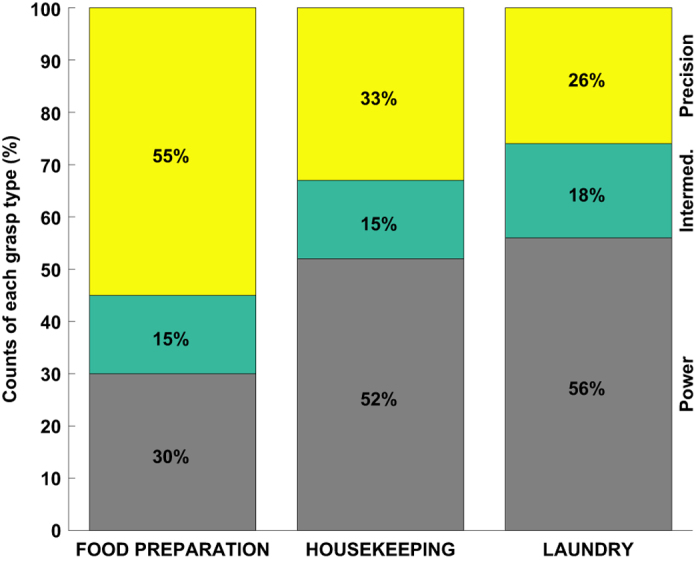
Power, Intermediate and Precision grasps comparison. Distribution of Power, Intermediate and Precision grasp types for each of the three types of ADLs.

The Feix taxonomy enables wider categorical analysis of grasps. Figure 3 shows the distribution of power, intermediate and precision grasps for specific DADLs. It can be seen that food preparation and laundry activities contrast in the proportion of precision and power grasps. For example, 56% of total grasp time in the laundry activities belongs to the power category revealing the extensive usage of iron. Oppositely, more than half of the grasps in food preparation are precision grasps such as pinch and prismatic configurations. The wide use of precision grasps for food preparation follows a similar trend to Vergara *et al*.^20^, where authors demonstrated that pinch grasp covered 54% of total food preparation duration. Finally, the distribution of grasps for housekeeping closely matches the results of Bullock^23^ (average of 55% for power and average of 33% for precision among two housekeepers in their results compared to 52 and 33% for power and precision types, respectively, in our work). Furthermore, 69 and 27% of the total number of grasps were performed with abducted and adducted thumb, respectively. Finally, 26, 18 and 52% of grasps fell into palm, side and pad opposition types; the remaining 4% of grasps belong to the non-prehensile group (lift and push), which cannot be classified by thumb position and opposition type according the Feix taxonomy.

### The use of top grasps by duration and frequency

The frequent use of IndFE, adducted thumb and medium wrap grasp types in our experiments is explained by the common use of power grasps engaging palm and five fingers with different thumb and finger configurations. This way, a wide set of objects of different sizes and shapes such as a knife, a vacuum cleaner holder, clothes, iron, etc. can be grasped. Similar patterns are observed in other studies. Specifically, medium wrap grasp constitutes around 11% of the total duration and around 6.5% of the total grasp count of our dataset. In the work of Bullock *et al*.^23^, the medium wrap is the most frequently used grasp with the duration and frequency proportions of approximately 23 and 14%, correspondingly. On the other hand, Vergara *et al*.^20^ employ the notion of the cylindrical grasp, which functionally resembles the medium wrap used in our work. In their work, cylindrical grasp is the fourth most used grasp with the total duration proportion of around 9%.

The dissimilarity in values is explained by the different taxonomies employed in the three studies, and the difference in the experimental activities and their durations. Different ADLs can inherently result in the dominant usage of either power or precision grasps. A suitable example is the work of a machinist, who systemically handles tools. On the contrary, food preparation tasks require more precise actions, hence the frequent use of precision grasp is expected. Bullock *et al*.^23^ conducted four experiments, comprised of housekeeping and machinist activities (two subjects for each category). Vergara *et al*.^20^ introduced a wider range of ADLs (eight types in total, where the tasks of our food preparation class fall into feeding, housekeeping and food preparation types).

Our data for housekeeping and laundry ADLs shows the dominance of the medium wrap grasp among all participants. The high duration and frequency proportion in use of the medium wrap in housekeeping (around 45% of the housekeeping duration and 20% of the frequency) and laundry (around 38% of the laundry duration and 30% of the frequency) activities is explained by the recurrent use of this grasp for holding an iron, clothes and a vacuum cleaner handle. These actions comprise a major duration proportion of these ADLs. In Bullock *et al*.^23^, average results for housekeepers’ use of medium wrap is 17.5% duration and 12.5% frequency proportions. Laundry activities in our study involve handling clothes and working with an iron, which implies high number of form closure cylindrical grasps (i.e. medium wrap grasps) and this is demonstrated by even higher frequency of medium wrap usage (around 30%) as compared to housekeeping activities. Results of our study and Bullock et al.^23^ clearly demonstrate the dominance of the form closure power grasps involving the palm and five fingers in housekeeping and laundry ADLs.

### Food preparation ADL comparison

Food preparation experiments generated three quarters of the total number of grasps in our dataset (see Table 1). The two highest duration grasps in food preparation are IndFE and writing tripod (around 19% and 14% of total duration, respectively). The outline and distribution of grasp duration and frequency by the two randomly selected subjects 1 and 3 look mostly similar with the exception of few cases. Subject 1 uses inferior pincer grasp frequently compared to the frequent use of lift by subject 3. The grasp with the highest duration in both subjects is the writing tripod. Writing tripod, a variation of the pinch, is a precision grasp employing three fingers. IndFE, on the other hand, is a power grasp, which engages palm with an adducted thumb. In Vergara *et al*.^20^, we observe the prevalence of pinch and intermediate power-precision (IntPP), with more than 40% and 19% of the total food preparation duration, respectively. IntPP in their taxonomy is described as the grasp which engages the palm and uses the thumb and index finger to stabilize an object, matching the definition of the IndFE in our work.

Despite being the top two grasps in food preparation, there is a notable discrepancy in the percentage of the usage of the grasps (19 and 14% for IndFE and writing tripod in our work as compared to the corresponding IntPP and pinch grasps with 19 and 40%, respectively). This discrepancy is due to the differences in experimental activities undertaken by subjects and the taxonomy. Specifically, food preparation consisted of selecting ingredients, preparing them to be cooked and cooking them^20^. In addition to these activities, our food preparation session contained food consumption and cleaning (e.g. wiping the table and washing the dishes), which notably diversified the set of grasps applied by the subjects. It is also important to note the narrower selection of grasps (9 in Vergara *et al*.^20^ versus 35 in our set), which would potentially lead to the generalization of similar grasps (e.g. variations of precision grasps).

Importantly, our work and Vergara *et al*.^20^ revealed frequent use of analogous grasp types in food preparation ADL, which supports the validity of our results.

### Housekeeping ADL comparison

**Figure 4:**
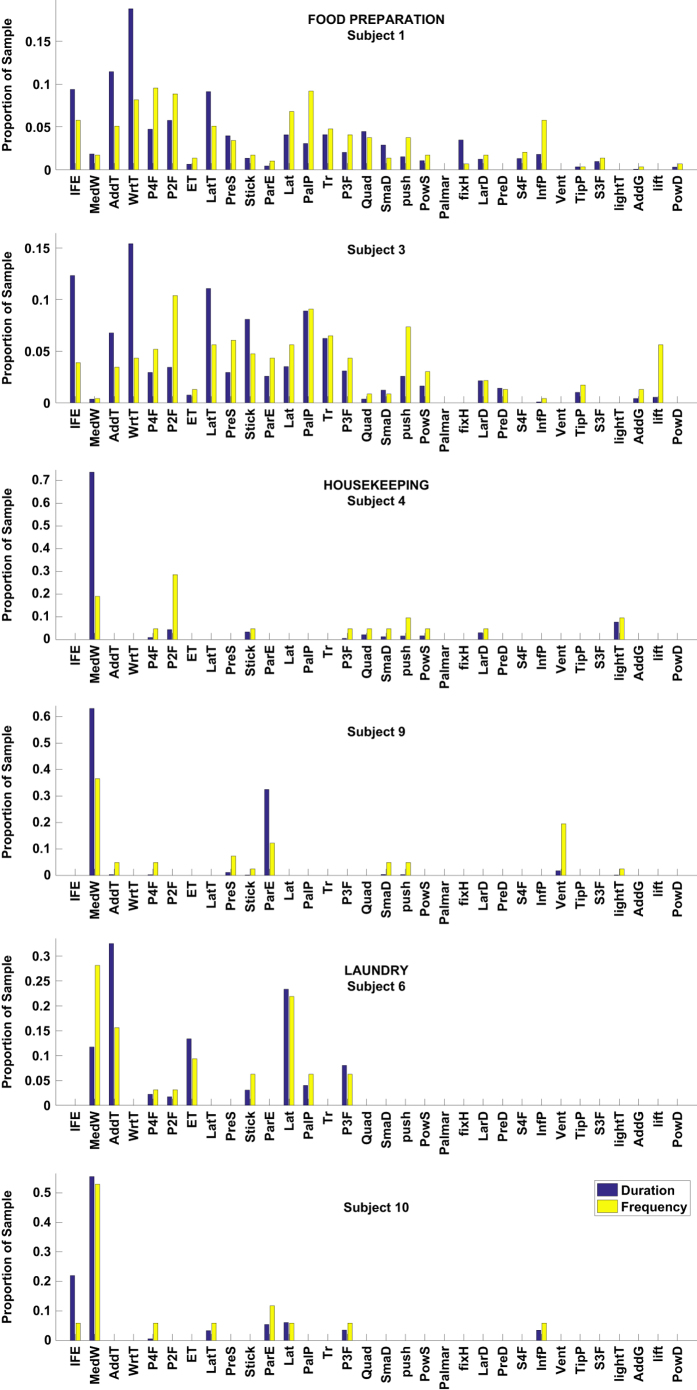
Comparative analysis of subjects’ performance. Relative distribution of grasp types durations and frequencies for two randomly selected human subjects in each type of activities of daily living.

Total duration of the experiments in subjects 4 and 9 differ significantly: 2978 and 1861 seconds, respectively. With longer experiment time, subject 4 applies a wider range of grasps. The difference can be observed in subject 4’s predominant use of the prismatic 4-finger and the light tool grasps with 30 and 10%, respectively, of the total grasp frequency in housekeeping ADL. Subject 9 rarely applied the aforementioned grasps (see Fig. 4 housekeeping subplot). Contrarily, subject 9 used ventral and parallel extension grasps with their count comprising around 20 and 10% of total grasp frequency count in this activity, respectively. The most distinct commonality between the two subjects’ grasp patterns is the strong prevalence of adducted thumb grasp with more than 60% of total duration for each participant. It can be also observed that subjects have similar results for usage statistics of precision 4-finger, stick, small diameter and push grasps.

### Laundry ADL comparison

Subjects 6 and 10 went through all ADLs with 2449 and 2184 seconds duration, respectively. Subject 10 finished the experiment about 4 min earlier, and their record contains less grasps. Figure 4 indicates several differences in the participants’ data. Specifically, subject 6 employed prismatic 2-finger, stick and palmar grasps for a notable duration, while subject 10 did not use these grasps for their tasks. On the contrary, lateral tripod, inferior pincer and IndFE were observed several times in subject 10, and rarely appear in subject 6. Additionally, subject 6 used the lateral grasp around four times more than subject 10 (around 21 and 5%, respectively). The variation can be explained by the difference in the set of performed tasks and the method of their execution. For instance, IndFE grasp is used by subject 10 more than 20% of the total grasp duration in laundry activity for operating an iron. The same operation was accomplished using adducted thumb grasp by subject 6 (more than 30% of total grasp duration in laundry). Usage of medium wrap, prismatic 4-finger and prismatic 3-finger grasps are nearly the same.

### Grasp frequency comparison between DADLs

**Figure 5:**
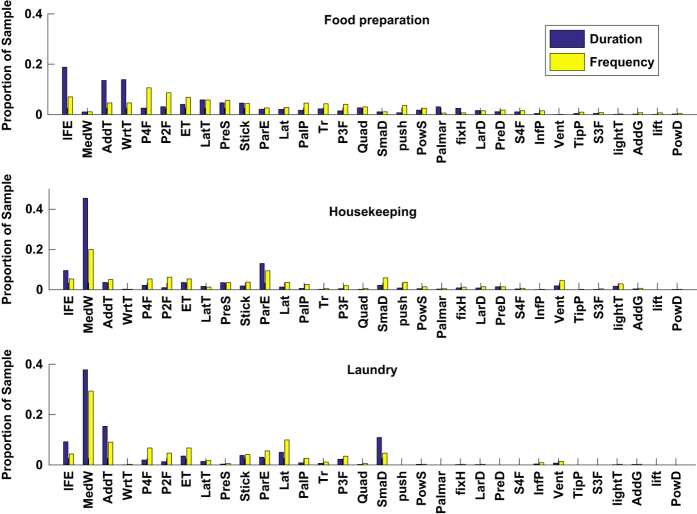
Relative grasp durations and frequencies across ADLs. Duration and frequency proportions for all grasps illustrated for food preparation, housekeeping and laundry tasks.

We also averaged total proportional durations and frequencies of grasps for each of the three DADLs across all subjects (see Fig. 5). The figure illustrates more frequent use of medium wrap and parallel extension in housekeeping and laundry DADLs, while precision grasps account for more than half of the food preparation grasps (around 55%). Distribution of grasp durations in housekeeping and laundry are more similar by the nature of tasks accomplished (e.g. operating appliances and manipulation with cloth).

## Additional information

**How to cite this article:** Saudabayev, A. *et al.* Human grasping database for activities of daily living with depth, color and kinematic data streams. *Sci. Data* 5:180101 doi: 10.1038/sdata.2018.101 (2018).

**Publisher’s note:** Springer Nature remains neutral with regard to jurisdictional claims in published maps and institutional affiliations.

## Supplementary Material



## Figures and Tables

**Table 1:** Duration and frequency of 32 grasps identified during experiments sorted by total duration.

#	**Grasp Name**	**Total**				**Food Preparation**				**Housekeeping**				**Laundry**			
		**Dur(s)**	**Dur(s), %**	**Freq**	**Freq, %**	**Dur(s)**	**Dur(s), %**	**Freq**	**Freq, %**	**Dur(s)**	**Dur(s), %**	**Freq**	**Freq, %**	**Dur(s)**	**Dur(s), %**	**Freq**	**Freq, %**
1	'index finger extension'	3804	16.60	251	6.56	3285	18.86	206	7.05	364	9.52	30	5.32	154	9.22	15	4.41
2	'medium wrap'	2557	11.16	245	6.4	188	1.08	32	1.1	1736	45.42	113	20.04	633	37.83	100	29.41
3	'adducted thumb'	2766	12.07	194	5.07	2370	13.6	135	4.62	139	3.63	28	4.96	257	15.36	31	9.12
4	'writing tripod'	2427	10.59	138	3.61	2416	13.87	136	4.65	10	0.27	1	0.18	1	0.05	1	0.29
5	'prismatic 4 finger'	570	2.49	363	9.49	454	2.61	310	10.61	83	2.16	30	5.32	33	1.98	23	6.76
6	'prismatic 2 finger'	609	2.66	305	7.97	547	3.14	254	8.69	40	1.04	35	6.21	22	1.34	16	4.71
7	'extension type'	904	3.95	252	6.59	708	4.06	199	6.81	136	3.56	30	5.32	60	3.59	23	6.76
8	'lateral tripod'	1106	4.82	183	4.78	1018	5.84	170	5.82	64	1.67	7	1.24	24	1.41	6	1.76
9	'precision sphere'	955	4.17	187	4.89	815	4.68	165	5.65	134	3.5	20	3.55	6	0.36	2	0.59
10	'stick'	930	4.06	163	4.26	795	4.56	128	4.38	71	1.87	21	3.72	64	3.81	14	4.12
11	'parallel extension'	918	4.01	150	3.92	370	2.12	78	2.67	497	13.01	53	9.4	52	3.08	19	5.59
12	'lateral'	500	2.18	138	3.61	363	2.09	83	2.84	52	1.36	21	3.72	85	5.07	34	10
13	'palmar pinch'	342	1.49	158	4.13	305	1.75	134	4.59	23	0.6	15	2.66	14	0.85	9	2.65
14	'tripod'	414	1.81	132	3.45	397	2.28	125	4.28	6	0.15	3	0.53	11	0.65	4	1.18
15	'prismatic 3 finger'	320	1.40	143	3.74	262	1.5	120	4.11	20	0.51	11	1.95	38	2.28	12	3.53
16	'quadpod'	480	2.09	95	2.48	470	2.7	90	3.08	7	0.19	3	0.53	3	0.18	2	0.59
17	'small diameter'	456	1.99	82	2.14	191	1.09	33	1.13	83	2.16	33	5.85	183	10.93	16	4.71
18	'push'	159	0.70	128	3.35	128	0.74	107	3.66	31	0.81	21	3.72	0	0	0	0
19	'power sphere'	335	1.46	83	2.17	311	1.79	74	2.53	20	0.51	8	1.42	4	0.24	1	0.29
20	'palmar'	556	2.43	21	0.55	545	3.13	18	0.62	11	0.3	3	0.53	0	0	0	0
21	'fixed hook'	471	2.05	28	0.73	434	2.49	20	0.68	34	0.88	7	1.24	2	0.14	1	0.29
22	'large diameter'	321	1.40	53	1.39	287	1.65	44	1.51	32	0.82	8	1.42	2	0.15	1	0.29
23	'precision disk'	257	1.12	60	1.57	202	1.16	52	1.78	55	1.45	8	1.42	0	0	0	0
24	'sphere 4 finger'	211	0.92	50	1.31	198	1.14	46	1.57	13	0.34	4	0.71	0	0	0	0
25	'inferior pincer'	86	0.37	49	1.28	78	0.45	45	1.54	1	0.02	1	0.18	7	0.42	3	0.88
26	'ventral'	97	0.42	34	0.89	10	0.06	3	0.1	73	1.92	26	4.61	13	0.78	5	1.47
27	'tip pinch'	79	0.35	29	0.76	79	0.45	28	0.96	1	0.01	1	0.18	0	0	0	0
28	'sphere 3 finger'	95	0.42	25	0.65	91	0.52	23	0.79	4	0.11	2	0.35	0	0	0	0
29	'light tool'	84	0.36	24	0.63	16	0.09	7	0.24	66	1.72	16	2.84	2	0.12	1	0.29
30	'adduction grip'	54	0.23	27	0.71	37	0.21	22	0.75	14	0.35	4	0.71	3	0.18	1	0.29
31	'lift'	12	0.05	21	0.55	12	0.07	21	0.72	0	0	0	0	0	0	0	0
32	'power disk'	46	0.20	15	0.39	41	0.23	14	0.48	5	0.13	1	0.18	0	0	0	0
	Total	22921		3826		17423		2922		3825		564		1673		340	
