# Erratum: Human grasping database for activities of daily living with depth, color and kinematic data streams

**DOI:** 10.1038/sdata.2018.143

**Published:** 2018-07-24

**Authors:** Artur Saudabayev, Zhanibek Rysbek, Raykhan Khassenova, Huseyin Atakan Varol

*Scientific Data* 5:180101 doi: 10.1038/sdata.2018.101 (2018), Published 29 May 2018; Updated 24 July 2018

An incorrect URL was erroneously included in Data Citation 1. The correct URL has now been inserted into the HTML and PDF versions.

